# Should We Pay for Our Social Media/Messenger Applications? Preliminary Data on the Acceptance of an Alternative to the Current Prevailing Data Business Model

**DOI:** 10.3389/fpsyg.2020.01415

**Published:** 2020-07-14

**Authors:** Cornelia Sindermann, Daria J. Kuss, Melina A. Throuvala, Mark D. Griffiths, Christian Montag

**Affiliations:** ^1^Department of Molecular Psychology, Institute of Psychology and Education, Ulm University, Ulm, Germany; ^2^Department of Psychology, Nottingham Trent University, Nottingham, United Kingdom

**Keywords:** data business model, surveillance capitalism, social media, big five, personality, payment model

## Abstract

In the age of surveillance capitalism, the prevailing business model underlying the use of social media applications (“apps”) foresees the exchange of personal data for the allowance to use an online service. Such a data business model comes with many potential negative side effects ranging from violation of privacy issues to election manipulation. Therefore, it is of utmost importance to think of alternatives to the current data business model. The present study investigated how strong the support would be for a monetary payment model among a sample of 210 participants. Participants were asked about their willingness to pay for social media, if in turn their data would be private and other problems concerning social media use would be tackled. Only one-fifth of participants (21.43%) supported such a model. From the Big Five personality traits, Agreeableness was positively associated with support of such a model. Finally, data are also provided on how much participants would be willing to pay for social media on a monthly basis. The present study’s findings are of a preliminary nature and will contribute to the start of an important discussion.

## Introduction

It has been estimated that 3.8 billion humans used social media^1^ and messenger services in 2020 (We Are Social et al., 2020). The most popular platforms in the Western part of the world derive from Facebook Inc. (We Are Social et al., 2020). Facebook owns not only the Facebook platform itself, but also Instagram and the messenger application WhatsApp. In the Eastern part of the world, in particular in China, WeChat dominates the market (Montag et al., 2018; We Are Social et al., 2020).

Although different social media platforms and messenger apps exist offering various functions and content to their users, the prevailing business model to earn money is the data business model. In short, users can use an online service in exchange for their data being used by the platform operators. Such digital footprint data are studied and analyzed by the social media and messenger app companies by means of algorithms, and the online profiles are sold to the marketing industry in order to enable them to engage in microtargeting (Kirkpatrick, 2016; Matz et al., 2020). Microtargeting entails sending a customized promotional message to an individual. In this regard, one study estimated that advertisers pay around $25 CPM (cost per mille; costs per 1,000 impressions) to reach an average user (but differences in the prices exist) (Papadopoulos et al., 2017). The data business model has been highly criticized because it raises ethical questions in the area of privacy and also encourages developers to design platforms which are “addictive” (Burt, 2019; Montag et al., 2019; for the addictive potential of platforms see Sha et al., 2019; Sindermann et al., 2020a, b). This is due to more time being spent on the platforms, leading to more user data being assessed, which in turn leads to better predictions of the users’ preferences through the algorithms used.

Finally, some of the elements of social media platforms, such as the newsfeed, are designed to show users what they like (based on the assumptions drawn from the digital footprints left on the platform) to make the users spending more time on the plaform (Montag et al., 2019). This personalization of content has several advantages, such as the “automatic” reduction of content that users are not interested in without the need for users to filter all the information available on their own. Still, benefits to the user are not clear, resulting in attenuation of privacy concerns (Aguirre et al., 2016). Personalization can also result in problems such as filter bubbles (Sindermann et al., 2020c). Of importance, personalized content (whether personalized by algorithms or by the users themselves) may be associated with radicalization and might even undermine democracy, especially if users decide to inform themselves about the political news exclusively via social media (Sunstein, 2004; Stroud, 2010; Bozdag and van den Hoven, 2015).

In the age of surveillance capitalism (Zuboff, 2015, 2019) it is of high importance to think of alternatives to the prevailing data business model because social media blurs the boundaries between the public and the private. By being inclusive, status-disregarding, discourse-generating, and theme-comprehensive, social media as virtual communities involve the entire spectrum of necessary preconditions for a public sphere (Habermas, 1998). Platforms such as Facebook and Instagram can be considered virtual communities (Rheingold, 1993) which perform the function of a public sphere, allowing individuals to come together to share experiences and opinions, driving democracy via freedom of speech and assembly (Habermas, 1998). Therefore, social media platforms are not merely *a* public sphere, but can be considered the *ideal* public sphere.

Given this important role of social media, it is questionable to what extent social media companies should provide this public sphere on their own, without other provisioning and regulatory bodies’ involvement (e.g., the European Union, other governmental institutions, and NGOs). Researchers have called for stronger regulation of social media companies (Griffiths et al., 2018). Moreover, the General Data Protection Regulation recently adopted in the European Union and the European Economic Area Regulation (EU 2016/679) is a first step in ensuring data privacy and additional measures need to be taken to give back control over users’ privacy rights. This may include replacing the data business model with a monetary payment model investigated in the present study. This would likely reduce the aforementioned negative side-effects of the data business model because in exchange for the allowance to use a social media service actual money is being paid (e.g., via a subscription model as successfully used by Netflix and Spotify; Netflix as cited in Statista, 2020; Spotify, 2020). As a consequence, companies such as Facebook would need to refrain from selling social media users’ data to the marketing industry.

Of note, several studies examining the willingness to pay for specific (personal) data exist (Carrascal et al., 2013; Egelman et al., 2013; Schreiner and Hess, 2015). For example, a study on web browsing reports that users value their online browsing history at around €7 (Carrascal et al., 2013). Another study by Schreiner and Hess (2015) reported that perceived usefulness and trust in a fictive premium version of Facebook with more privacy protection functions positively influenced how much participants were willing to pay for this fictive premium version of Facebook. However, within and across studies it is important to note that privacy behavior must be seen as a contextual phenomenon (Acquisti et al., 2013; Carrascal et al., 2013; Morando et al., 2014) dependent among others to an extent on how relevant, trust-worthy, value-added, and engaging personalization is currently perceived by users (Aguirre et al., 2016).

Based on this background literature, the present study aimed to answer two specific questions: (i) what proportion of users would support such an alternative business/monetary subscription model for social media offers and messenger apps, and (ii) do specific sociodemographic and personality variables predict support for such a model (numerous studies report associations of demographics and personality with various social media use variables; Montag et al., 2015; Kuss and Griffiths, 2017; Sindermann et al., 2020a). The study is necessarily of an exploratory nature. Therefore, no hypotheses were formulated given the scarce literature on this topic.

## Materials and Methods

### Procedure and Sample

The present dataset was collected via an online survey investigating several research questions dealing with the topics of smartphone use, social media use, and news consumption (programmed with the Survey Coder platform^2^^3^). The link to the study was advertised offline (e.g., on television and radio) as well as online (e.g., via social media and websites of news agencies) and participation was voluntary. Therefore, the participants in the present study form a convenience sample. As an incentive, participants of the study received anonymous feedback, for example on their scores on the Big Five of Personality in comparison to the average scores of the other participants of the study. All participants provided informed consent prior to participation. The study followed the guidelines of the German Society for Online Research^4^.

After data cleaning (see Supplementary Material), the final sample comprised *N* = 210 participants (*n* = 117 males; *n* = 91 females; *n* = 2 “third gender”). The mean age of the sample was 35.82 years (*SD* = 12.30 years; median = 33.50 years) with a range from 18 to 73 years. Approximately half of the participants indicated university degree as their highest educational degree (*n* = 108). The other participants stated a university of applied sciences degree (*n* = 22), or some kind of school degree (*n* = 80) as their highest educational degree.

### Materials

#### Big Five Inventory

To assess the Big Five of Personality, the German version of the Big Five Inventory (BFI) was applied (Rammstedt and Danner, 2017). It comprises 45 items answered on a five-point Likert-Scale from 1 (“very inapplicable”) to 5 (“very applicable”). The 45th item concerning disputes with others (which is unique to the German version of this instrument) was not included in the final analyses to allow for closer comparability with other studies. Despite the possibility of calculating subscale scores of each broad Big Five factor to assess sub-facets, the present work will focus on the broad Big Five factors. The internal consistencies (Cronbach’s alphas) of the five scales in the present sample were 0.82, 0.83, 0.88, 0.75, and 0.86 for Openness, Conscientiousness, Extraversion, Agreeableness, and Neuroticism, respectively.

#### Willingness to Pay for Social Media/Messenger Services

The willingness to pay for social media/messenger services was assessed in two ways. First of all, four items on the willingness to pay a monthly usage fee for social media (such as Facebook and Instagram) were assessed. These included the willingness to pay (i) “if thereby it is ensured that my data accrued there are not used for marketing purposes,” (ii) “if thereby it is ensured that my data accrued there are better protected,” (iii) “if thereby it is ensured that the social media offers are designed in a way that does not aim to prolong the time users spend online,” (iv) “if thereby it is ensured that the problem of fake news and radicalization is reduced.” Each item was answered on a five-point Likert-Scale from 1 (“strongly disagree”) to 5 (“strongly agree”). The scores in the four items were collapsed into one aggregate scale with an internal consistency (Cronbach’s alpha) of 0.89. For the results of a principal component analysis, please see Supplementary Material. The items are presented in English and German language in Tables 1, 2. The German version was used in the present study.

**TABLE 1:** Questionnaire assessing the willingness to pay for social media (WtP-SM) in English language.

I am willing to pay a monthly usage fee (money) for social media services such as Facebook or Instagram, if thereby it is ensured that my data accrued there are not used for marketing purposes.
I am willing to pay a monthly usage fee (money) for social media services such as Facebook or Instagram, if thereby it is ensured that my data accrued there are better protected.
I am willing to pay a monthly usage fee (money) for social media services such as Facebook or Instagram, if thereby it is ensured that the social media offers are designed in a way that does not aim to prolong the time users spend online.
I am willing to pay a monthly usage fee (money) for social media services such as Facebook or Instagram, if thereby it is ensured that the problem of fake news and radicalization is reduced.

*The items are answered on the following response scale: “strongly disagree”, “disagree”, “neither agree nor disagree”, “agree”, “strongly agree”.*

**TABLE 2:** Questionnaire assessing the willingness to pay for social media (WtP-SM) in German language.

Ich bin bereit für Social Media Angebote wie beispielsweise Facebook oder Instagram pro Monat eine Nutzungsgebühr (Geld) zu bezahlen, wenn dadurch sichergestellt wird, dass meine dort anfallenden Daten nicht für Marketing-Zwecke genutzt werden.
Ich bin bereit für Social Media Angebote wie beispielsweise Facebook oder Instagram pro Monat eine Nutzungsgebühr (Geld) zu bezahlen, wenn dadurch sichergestellt wird, dass meine dort anfallenden Daten besser geschützt werden.
Ich bin bereit für Social Media Angebote wie beispielsweise Facebook oder Instagram pro Monat eine Nutzungsgebühr (Geld) zu bezahlen, wenn dadurch sichergestellt wird, dass die Social Media Angebote so gestaltet sind, dass sie nicht auf die Verlängerung der Online-Zeiten der Nutzer abzielen.
Ich bin bereit für Social Media Angebote wie beispielsweise Facebook oder Instagram pro Monat eine Nutzungsgebühr (Geld) zu bezahlen, wenn dadurch sichergestellt wird, dass das Problem der Fake News und Radikalisierung reduziert wird.

*The items are answered on the following response scale: “stimme überhaupt nicht zu”, “stimme nicht zu”, “weder noch”, “stimme zu”, “stimme absolut zu”.*

Additionally, participants were asked to indicate how much money (in Euros) they were willing to pay per month for a single social media service such as Facebook and Instagram. The same question was also asked about paying for a single messenger service.

### Statistical Analyses

First, descriptive statistics of all variables of interest were calculated. The skewness and kurtosis of all Big Five scales and the aggregate score on whether participants were willing to pay for social media (such as Facebook and Instagram) were below ±1, indicating a normal distribution (Miles and Shevlin, 2001). However, the skewness and kurtosis of the two items asking about how much participants would be willing to pay per month were much higher. Therefore, boxplots of these variables were inspected and some univariate outliers were identified. More specifically, scores outside the boxplot whiskers (set according to the formula by Tukey (1977): {25th-Quantile − [1.5 × (75th-Quantile − 25th-Quantile)]} and {75th-Quantile + [1.5 × (75th-Quantile − 25th-Quantile)]}) were identified as outliers.

For these participants (in total *n* = 7), the actual response was replaced by the highest score, which was still identified as non-outlier (i.e., €12.50). Of note, completely excluding the seven participants from the analyses did not markedly change the main results. However, it slightly reduced the mean values of the two items on how much participants were willing to pay. Moreover, it reduced effect sizes and increased *p*-values of some correlations between personality and payment variables. For example, the correlation between Agreeableness and the aggregate score on whether participants were willing to pay for social media (such as Facebook and Instagram) was *r* = 0.17 and *p* = 0.016 in the sample of 203 participants. After dealing with the outliers, the skewness and kurtosis of both variables in parts still exceeded ±1. Exceeding a value of ±1 indicates a violation of the normal distribution assumption (Miles and Shevlin, 2001). This was also underlined by significant values utilizing Shapiro–Wilk tests (although this test is biased towards significance due to the sample size). Finally, the histograms clearly indicated a non-normal distribution (see Supplementary Material). Consequently, non-parametric analyses were used to investigate these two items, and parametric analyses for the remaining tests.

Associations of all variables of interest with age and gender were calculated. Pearson’s correlations were used to calculate correlations of age with the Big Five and the aggregate score on whether participants were willing to pay for social media (such as Facebook and Instagram) (see aforementioned assumption of a normal distribution). Spearman’s correlations were calculated for the associations of age with the two items asking about how much participants would be willing to pay per month (see aforementioned violation of criteria to assume a normal distribution). To examine gender differences in the Big Five and the aggregate score on whether participants were willing to pay for social media (such as Facebook and Instagram), *t*-tests were calculated (Welch’s *t*-tests if equal variances could not be assumed based on a Levene’s test; see aforementioned assumption of a normal distribution). Gender differences in the two items asking about how much participants would be willing to pay per month were investigated by Mann–Whitney *U*-tests (see aforementioned violation of criteria to assume a normal distribution).

Finally, correlations between personality and the aggregate score on whether participants were willing to pay for social media were calculated by means of Pearson’s correlations corrected for age (see section “Results”). To examine the associations between personality and how much money participants would be willing to pay for social media (such as Facebook and Instagram), and messenger services per month, Spearman’s correlations corrected for age were calculated. All correlations are presented for the total sample and for males and females separately. Due to the low number of participants stating “third gender” as their designated gender identity, no separate results for this group are presented.

## Results

### Descriptive Statistics, Associations With Age, and Gender Differences

**FIGURE 1:**
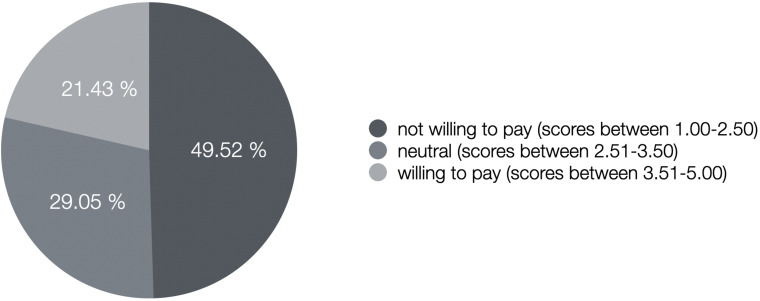
Proportion of participants who were not willing to pay on average (scores 1.00–2.50), were neutral (scores 2.51–3.50), or were willing to pay (scores 3.51–5.00) for social media (such as Facebook and Instagram) based on the aggregate score of the four items.

Descriptive statistics are presented in Table 3. Moreover, Figure 1 shows the proportion of participants who were not willing to pay (scores 1.00–2.50), were neutral (scores 2.51–3.50), or were willing to pay (scores 3.51–5.00) according to the aggregate score.

**TABLE 3:** Descriptive statistics of all variables of interest and differences between males and females.

	Total sample (*N* = 210)		Males (*n* = 117)	Females (*n* = 91)	Differences between males and females
	*M (SD)*	*Median*	*M (SD)*	*M (SD)*	
Openness	3.79 (0.64)	3.80	3.80 (0.63)	3.77 (0.66)	*t*(206) = 0.30, *p* = 0.766, *d* = 0.04
Conscientiousness	3.40 (0.66)	3.33	3.32 (0.60)	3.49 (0.73)	*t*(172.91) = −1.71, *p* = 0.089, *d* = 0.24
Extraversion	3.28 (0.84)	3.38	3.17 (0.85)	3.41 (0.82)	*t*(206) = −2.08, *p* = 0.039, *d* = 0.29
Agreeableness	3.58 (0.59)	3.67	3.55 (0.55)	3.60 (0.64)	*t*(206) = −0.67, *p* = 0.502, *d* = 0.09
Neuroticism	2.70 (0.79)	2.75	2.52 (0.76)	2.93 (0.79)	*t*(206) = −3.81, *p* < 0.001, *d* = 0.53
Willingness to pay	2.65 (1.13)	2.75	2.58 (1.12)	2.73 (1.14)	*t*(206) = −0.95, *p* = 0.344, *d* = 0.13
Amount of monthly social media fee	2.22 (2.96)	1.00	2.15 (2.95)	2.36 (3.01)	*W* = 5,059.5, *p* = 0.520, *r* = −0.04
Amount of monthly messenger fee	2.40 (2.73)	1.25	2.59 (2.84)	2.20 (2.59)	*W* = 5,742.5, *p* = 0.320, *r* = 0.07

*Willingness to pay: aggregate score of four items on whether participants were willing to pay for social media (such as Facebook and Instagram); amount of monthly social media fee: how much money participants would be willing to pay for one social media service, such as Facebook and Instagram, per month; amount of monthly messenger fee: how much money participants would be willing to pay for one messenger service per month. Two participants stated “third gender” as their gender identity and are not included in this table due to the low number of individuals in this group; *d* = Cohen’s D; *r* = effect size for Mann-Whitney *U*-tests.*

Age correlated significantly with Openness (*r* = 0.16, *p* = 0.25), Conscientiousness (*r* = 0.19, *p* = 0.007), and Neuroticism (*r* = −0.15, *p* = 0.029), as well as with the amount of money participants were willing to pay per month for a messenger service (*r*_*s*_ = −0.15, *p* = 0.027). Gender differences were found in Extraversion and Neuroticism only. Females had higher scores than males in both scales (see Table 3).

### Correlations Between Personality and Willingness to Pay for Social Media/Messenger Services

As can be seen in Table 4, the only significant associations were found between Agreeableness and the willingness to pay for social media (such as Facebook and Instagram) and the amount of money participants would be willing to pay for a messenger service. These correlations were positive and the effect sizes were rather small. The association between Agreeableness and the amount of money participants were willing to pay for social media (such as Facebook and Instagram) just failed to be statistically significant.

**TABLE 4:** Partial correlations between the Big Five personality traits and the items on the willingness to pay for social media and messenger services in the total sample.

	Total sample (*N* = 210)		
	Willingness to pay	Amount of monthly social media fee	Amount of monthly messenger fee
Openness	*r* = 0.11, *p* = 0.098	*r_*s*_* = 0.09, *p* = 0.175	*r_*s*_* = −0.03, *p* = 0.616
Conscientiousness	*r* = −0.08, *p* = 0.280	*r_*s*_* = −0.04, *p* = 0.592	*r_*s*_* = −0.05, *p* = 0.477
Extraversion	*r* = −0.01, *p* = 0.846	*r_*s*_* = −0.01. *p* = 0.907	*r_*s*_* = −0.07, *p* = 0.288
Agreeableness	*r* = 0.20, *p* = 0.004	*r_*s*_* = 0.13, *p* = 0.053	*r_*s*_* = 0.16, *p* = 0.023
Neuroticism	*r* = −0.02, *p* = 0.805	*r_*s*_* = −0.05, *p* = 0.495	*r_*s*_* = −0.06, *p* = 0.409

*Willingness to pay: aggregate score of four items on whether participants were willing to pay for social media (such as Facebook and Instagram); amount of monthly social media fee: how much money participants would be willing to pay for one social media service, such as Facebook and Instagram, per month; amount of monthly messenger fee: how much money participants would be willing to pay for one messenger service per month; all correlations presented are corrected for age.*

Investigating the same associations among males and females separately (Table 5) showed similar results among males. More specifically, moderate positive associations between Agreeableness and all variables on the willingness to pay for social media and messenger services were found among males only. Among females, all associations were non-significant.

**TABLE 5:** Partial correlations between the Big Five personality traits and the items on the willingness to pay for social media and messenger services among males and females.

	Males (*n* = 117)			Females (*n* = 91)		
	Willingness to pay	Amount of monthly social media fee	Amount of monthly messenger fee	Willingness to pay	Amount of monthly social media fee	Amount of monthly messenger fee
Openness	*r* = 0.12, *p* = 0.208	*r_*s*_* = 0.10, *p* = 0.281	*r_*s*_* = −0.08, *p* = 0.406	*r* = 0.11 *p* = 0.307	*r_*s*_* = 0.09, *p* = 0.386	*r_*s*_* = 0.04, *p* = 0.687
Conscientiousness	*r* = −0.10, *p* = 0.301	*r_*s*_* = −0.03, *p* = 0.721	*r_*s*_* = −0.22, *p* = 0.018	*r* = −0.06, *p* = 0.570	*r_*s*_* = −0.06, *p* = 0.583	*r_*s*_* = 0.15, *p* = 0.160
Extraversion	*r* = 0.04, *p* = 0.639	*r_*s*_* = 0.03, *p* = 0.740	*r_*s*_* = −0.01, *p* = 0.912	*r* = −0.12, *p* = 0.268	*r_*s*_* = −0.09, *p* = 0.409	*r_*s*_* = −0.13, *p* = 0.221
Agreeableness	*r* = 0.21, *p* = 0.024	*r_*s*_* = 0.25, *p* = 0.006	*r_*s*_* = 0.26, *p* = 0.004	*r* = 0.20, *p* = 0.064	*r_*s*_* = −0.01, *p* = 0.903	*r_*s*_* = 0.07, *p* = 0.493
Neuroticism	*r* = −0.00, *p* = 0.976	*r_*s*_* = −0.04, *p* = 0.632	*r_*s*_* = 0.03, *p* = 0.716	*r* = −0.06, *p* = 0.551	*r_*s*_* = −0.10, *p* = 0.368	*r_*s*_* = −0.17, *p* = 0.101

*Willingness to pay: aggregate score of four items on whether participants were willing to pay for social media (such as Facebook and Instagram); amount of monthly social media fee: how much money participants would be willing to pay for one social media service, such as Facebook and Instagram, per month; amount of monthly messenger fee: how much money participants would be willing to pay for one messenger service per month. Two participants stated “third gender” as their gender identity and are not included in this table due to the low number of individuals in this group; all correlations presented are corrected for age.*

When applying a very strict Bonferroni correction, none of the correlations remained significant (e.g., 0.05/15 = 0.003; because the Big Five scales were associated with three scales/items on the willingness to pay for social media/messenger services). However, this is a strict correction procedure and it should be noted that the rather small present sample size (especially when split by gender) had an impact on statistical significance.

## Discussion

The present study examined how strong a social media user’s support would be for an alternative model to the data business model which is currently being used by almost all social media companies. Beyond obtaining first insights into such support, the study also aimed to understand which socio-demographic variables and personality traits predicted support for such an alternative (i.e., paying for an online service with money).

In the present sample, approximately one-fifth of the participants (21.43%) stated they were willing to pay money (e.g., via a monthly subscription fee) for a social media online service. Of the participants, 29.05% were indecisive, whereas nearly half of the users stated that they were not willing to pay for such a service. This shows that paying money for social media in order to heighten privacy standards and reduce related problems such as radicalization finds no acceptance in a fairly large proportion of our investigated sample. It could be that either the majority of social media users (i) really do not care about privacy implications and just want to continue having free access to social media sites, or (ii) do not understand how their data is being used and/or exploited. Of additional interest, Supplementary Table S3 shows the mean values of the single items asking participants about their willingness to pay in order to reduce the use of their data for marketing purposes, to ensure better data protection, to reduce prolongation of users’ time spent online, and to reduce problems of fake news and radicalization. It turns out that individuals in the present sample were willing to pay most in order to reduce risks such as fake news and radicalization followed by paying for higher data protection, decreased use of data for marketing purposes, and decreased prolongation of online time, in ascending order.

If the identified percentages on the (un)willingness to pay for social media services are replicated in large representative studies, measures need to be taken to ensure data protection on social media sites. One of the recommendations would be to create large campaigns which explain the value of privacy, and to support a monetary alternative business model. Otherwise, new alternatives must be thought of. Giving users the option to choose between a monetary payment option (to heighten privacy) and the data payment option might be such an alternative. However, adoption of such an approach will disadvantage individuals on lower incomes who may not have the financial means to pay for such subscriptions. The present research shows that the search for alternative business models to the prevailing data business models is just beginning and represents an important and timely research endeavor. New solutions are urgently needed.

A second aim of the present study was to understand if specific demographic and personality characteristics predict stronger support for a monetary payment model in the realm of social media use. Whereas age and gender played minor roles in predicting such support, higher Agreeableness was positively associated with support for a monetary payment model. Whilst among both males and females Agreeableness was positively associated with the variable *willingness to pay for social media* (association in females just failed to be significant but effect sizes are similar), Agreeableness was also positively correlated with the proposed monthly fee for the social media/messenger service only among males. However, these observations need to be replicated in other samples.

Although messenger apps such as WhatsApp can be considered part of the umbrella term social media (Kuss and Griffiths, 2017) participants were specifically asked how much money they would pay for a service such as Instagram versus WhatsApp. In line with the very large distribution of WhatsApp (currently 1.5 billion users vs. 1 billion Instagram users), participants indicated a slightly higher monthly fee they were willing to pay for messenger services compared to social media, such as Facebook and Instagram (although this might also be a function of age). In 2019, Facebook earned $29.25 per user (at the moment of writing about €26.94; Facebook annual report as cited in Statista, 2020). Accordingly, roughly €2.50 per month would be required per user based on a subscription fee model to earn the same 2019 revenue. This number closely matches the mean provided by the participants of our sample (€2.22 for social media vs. €2.40 for a messenger service). From this perspective, the finding provides a good basis for discussions about a monetary payment model for social media/messenger services.

However, the present study’s findings (despite methodological limitations analyzed below) demonstrate that monetizing a currently free service (or offering a subscription-based alternative one) in return for data privacy appears to have a weak base of support. Based on Social Exchange Theory (Thibaut and Kelley, 1959) purporting a cost-benefits analysis in driving human decisions, behaviors, and expectations, it could be hypothesized that the current perceived benefits of social media platforms and app services outweigh the potential negatives (e.g., privacy concerns and echo chambers) (Lowry et al., 2011) and therefore users are reluctant to endorse a subscription-based model, solely on the proposition of safeguarding data privacy.

Instead, a monetized subscription-based business model offering commercial-free and data protected social exchanges could potentially find greater support if social media operators and regulators went beyond safeguarding data protection and rights of minors, into encouraging a more socially responsible social media business model. Equally with corporate social responsibility (CSR) initiatives in other industries – i.e., the food and beverage industry, which has endorsed healthier eating habits or more environmentally friendly agricultural and trading conditions (World Health Organization, 2004) – the greater promotion of user rights and representation, the reduction of poverty, social inequality, and greater access to media literacy, the prevention of sedentary lifestyles and obesity, radicalization, and the encouragement of social entrepreneurship could be just a few of the social media industry’s CSR initiatives for a healthier platform ecosystem (Afridi and Joseph Rowntree Foundation, 2011; Paus-Hasebrink et al., 2019; Throuvala et al., 2020).

Governmental interventions in this direction have started to be enacted (Department for Digital Culture Media Sport, 2019) in line with mounting social pressures for greater accountability in some countries (5Rights Foundation, 2019; Information Commissioner’s Office, 2019). Such measures would then render more tangible benefits to users, tapping into their basic psychological needs for autonomy, competence, and relatedness (Deci and Ryan, 1985). Additionally, an advertising-free, data protected and more socially responsible social media environment would potentially safeguard against technological burn-out (Peterka-Bonetta et al., 2019) triggered by information overload and impacting daily productivity (Duke and Montag, 2017; Rozgonjuk et al., 2020).

Higher regulation could also lead to diminished environmental/design triggers (“chasing” likes, followers), reinforcing prolonged user engagement and driving needs to control self-representation, content and relationships online (Throuvala et al., 2019a, b). Initiatives such as Instagram’s trial to ban “likes” (Griffin, 2019) are steps in the right direction, but need to be followed by robust and more socially inclusive policies. If CSR benefits are appropriately channeled to end users, it is likely that an alternative monetary model offering data protection and CSR will be more convincing and widely accepted. This reflects evidence suggesting that a higher value is ascribed to more positive experiences vs. references to money, which are usually negative due to the reminder of the cost to acquire a product or service rather than the pleasure or benefit of using it (Mogilner and Aaker, 2009). Future studies may therefore examine further the feasibility of a more integrative monetary business model.

Finally, it is worth drawing attention to the privacy paradox. This paradox describes the tendency to not protect one’s privacy (e.g., to disclose personal data) despite being concerned over one’s privacy (Norberg et al., 2007). For example, a study by Woodruff et al. (2014) investigated associations between generic privacy attitudes and responses to several hypothetical scenarios and outcomes. The study found that individuals categorized as privacy fundamentalists (high concern), pragmatists (mixed concern), and unconcerned (no/little concern) did not differ in their likelihood to disclose data in any of the scenarios or depending on outcome. Although the present study did not directly assess privacy concerns or actual behavior, one can interpret the willingness to pay as intention for a specific privacy protective behavior, while acknowledging a potential hypothetical bias between willingness to pay and users’ real intentions (Schmidt and Bijmolt, 2020). Therefore, whether individuals would ultimately actually pay for social media or messenger services remains debatable (for more information on the privacy paradox, see the review by Kokolakis, 2017).

The present study has several limitations, which need to be addressed. As mentioned, the study is not of representative nature and the sample size is rather small. This is also one of the reasons why the analyses were not run separately for the groups with different educational background (in addition to separately for gender). Another reason for this decision was that the level of education was unequally distributed in the present sample. Therefore, larger studies need to be conducted in this area. The study is viewed by the authors as a first step to start an important discussion in this area. Beyond this, the study is correlational in nature. Therefore, no causal interpretation between the variables is possible. The data were also of a self-report nature, therefore common method biases (such as social desirability) may have influenced the findings. Moreover, the results may also differ depending on geographical location, including Asia, where average earnings may be lower in comparison to Germany, and the respective sociocultural context may impact the results. In addition, many more factors putatively associated with the willingness to pay for social media services should be taken into account in future studies (Malhotra et al., 2004). For example, willingness to pay may depend upon whether individuals need to use social media platforms in the work context and upon an individual’s income. Finally, the effects of an option to actively allow big technology companies to use one’s data (i.e., by a “pay by sharing your data” option) should be further investigated in future studies. An additional item in a future survey would be to ask if individuals would be willing to pay to hinder third party apps to collect data about the user of a social media platform, although such apps might be helpful to run surveys such as the present one (Kalimeri et al., 2020; but see also differences across social media user groups as reported in Marengo et al., 2020).

## Data Availability Statement

All datasets present in this study are available at the Open Science Framework: https://osf.io/wehmj/. Moreover the datasets are uploaded alongside this article in the Supplementary Material (Supplementary Table S1).

## Ethics Statement

Ethical review and approval was not required for the study on human participants in accordance with the local legislation and institutional requirements. The participants provided their electronic informed consent to participate in this study prior to participation.

## Author Contributions

CM and CS designed the present work. CM drafted the first version of the Introduction and Discussion. CS wrote the Methods and Results, and conducted the statistical analysis, which were independently checked by CM. DK, MT, and MG critically revised the first draft of the manuscript. All authors approved the final version of this work.

## Conflict of Interest

The authors declare that the research was conducted in the absence of any commercial or financial relationships that could be construed as a potential conflict of interest.

## Footnotes

1 Of note, despite existing definitions, for the present work the term social media is used to explicitly refer to platforms such as Facebook and Instagram, whereas messenger apps/services refer to instant messenger services such as WhatsApp (Howard and Parks, 2012; Carr and Hayes, 2015).

2 https://www.surveycoder.com/,

3 https://ckannen.com/

4 https://rat-marktforschung.de/fileadmin/user_upload/pdf/R08_RDMS.pdf
