# Supplementary material for: Should We Pay for Our Social Media/Messenger Applications? Preliminary Data on the Acceptance of an Alternative to the Current Prevailing Data Business Model
Source: Front Psychol. 2020 Jul 14;11:1415. doi: 10.3389/fpsyg.2020.01415 (PMC7371851; doi:10.3389/fpsyg.2020.01415)
Supplement: Supplementary file 1 [file Presentation_1.pdf]

Supplementary Material

1 Data Cleaning

A total of 367 complete datasets were collected. Participants below the age of 18 were excluded from final analyses (*n*=12) because participation was only allowed from the age of 18. Additionally, participants indicating being older than 100 years were excluded (*n*=1). Finally, *n*=1 participant was excluded due to choosing the same response option throughout all items of the Big Five Inventory indicating careless responding. This leads to an effective sample size of *N*=353 (*n*=215 men; *n*=136 women; *n*=2 “third gender”) participants. However, several participants stated they did not use social media platforms such as Facebook or Instagram (*n*=106), or that they did not use messenger services (*n*=8), or that they did neither use social media nor messenger services (*n*=29). We decided to exclude these participants because asking a person about whether and how much he/she would be willing to pay for a service he/she does not use seemed not valid. Therefore, the final sample consisted of *N*=210 (*n*=117 men; *n*=91 women; *n*=2 “third gender”) participants.

2 Principal Component Analysis on the Four Items about whether Participants were Willing to Pay for Social Media

The Principal Component Analysis revealed one eigenvalue higher than 1 (eigenvalue = 3.05). The scree plot is shown in Supplementary Figure 1.

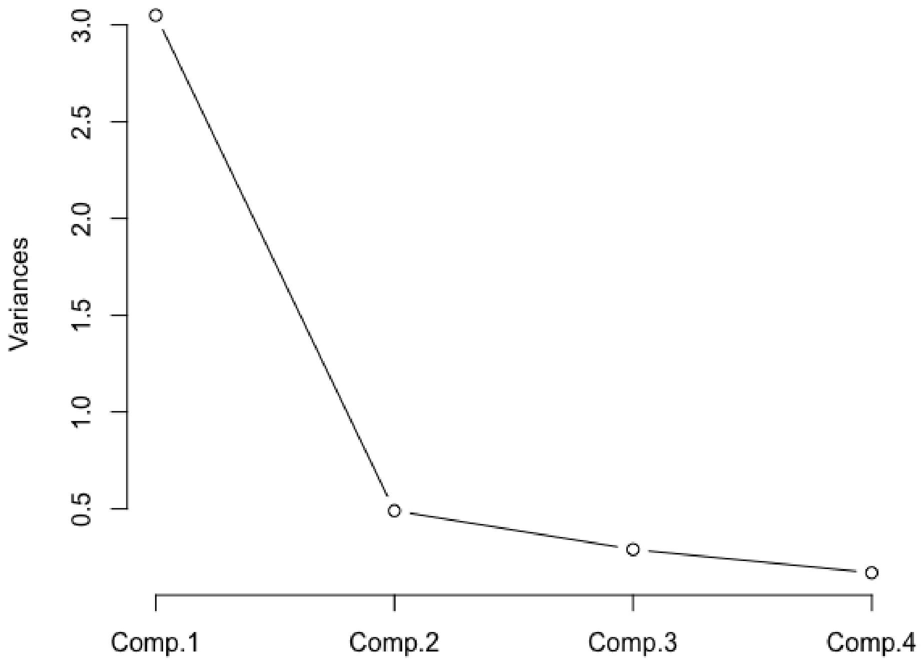

Supplementary Figure 1. Scree plot on the four items on the willingness to pay for social media i) “if thereby it is ensured that my data accrued there are not used for marketing purposes”, ii) “if thereby it is ensured that my data accrued there are better protected”, iii) “if thereby it is ensured that the social media offers are designed in a way that does not aim to prolong the time users spend online”, iv) “if thereby it is ensured that the problem of fake news and radicalization is reduced”.

3 Distributions of Responses to the Items on How Much Participants would be Willing to Pay for Social Media / Messenger Services

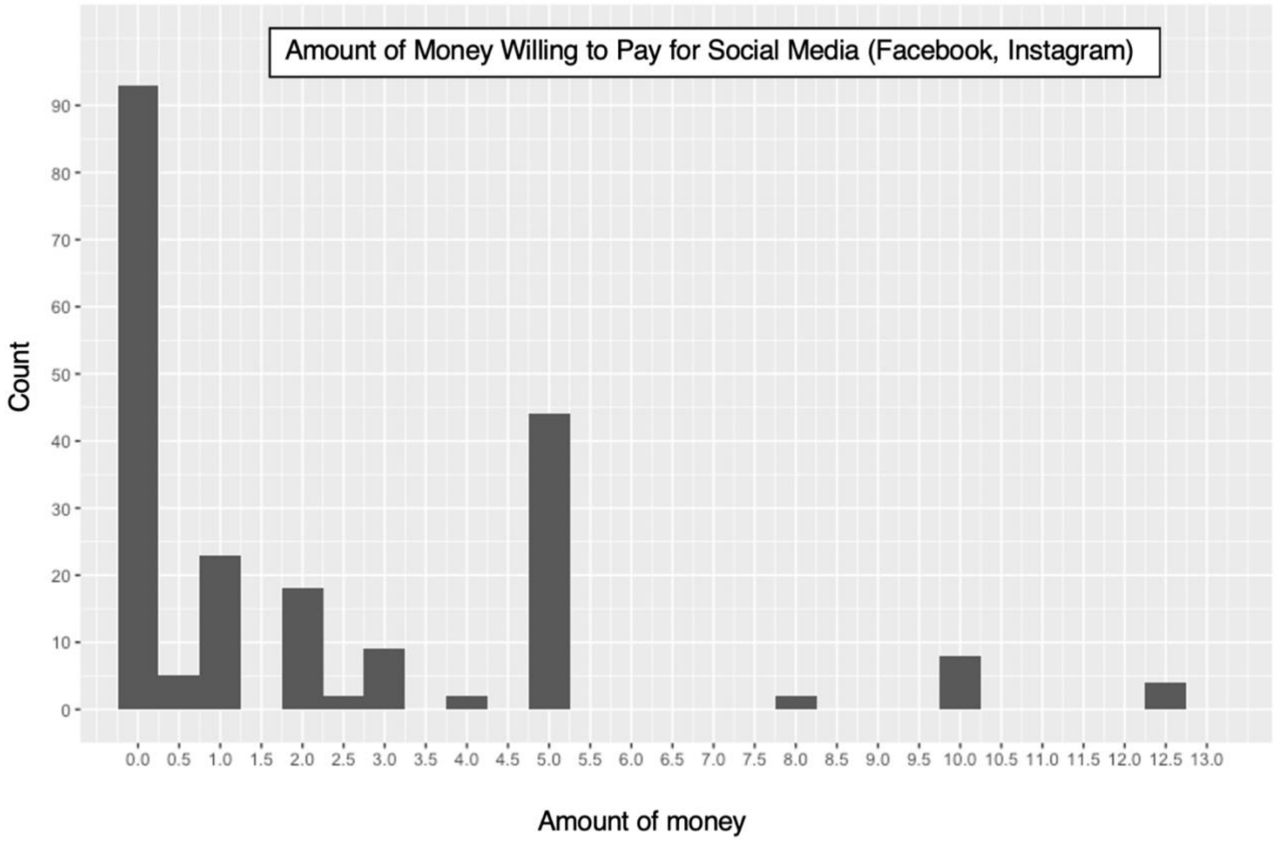

Supplementary Figure 2. Histogram of the variable on how much money participants would be willing to pay per month for a single social media (Facebook, Instagram) service; the x-axis shows the amount of money and the y-axis the number of participants indicating to be willing to pay the respective amount of money (after dealing with univariate outliers).

Supplementary Table 1.
Distribution of how much money participants would be willing to pay per month for a single social media (Facebook, Instagram) service (after dealing with univariate outliers)

€	0.00	0.30	0.50	1.00	2.00	2.50	3.00	3.80	4.00	4.99	5.00	8.00	10.00	12.50
count	93	1	4	23	18	2	9	1	1	1	43	2	8	4

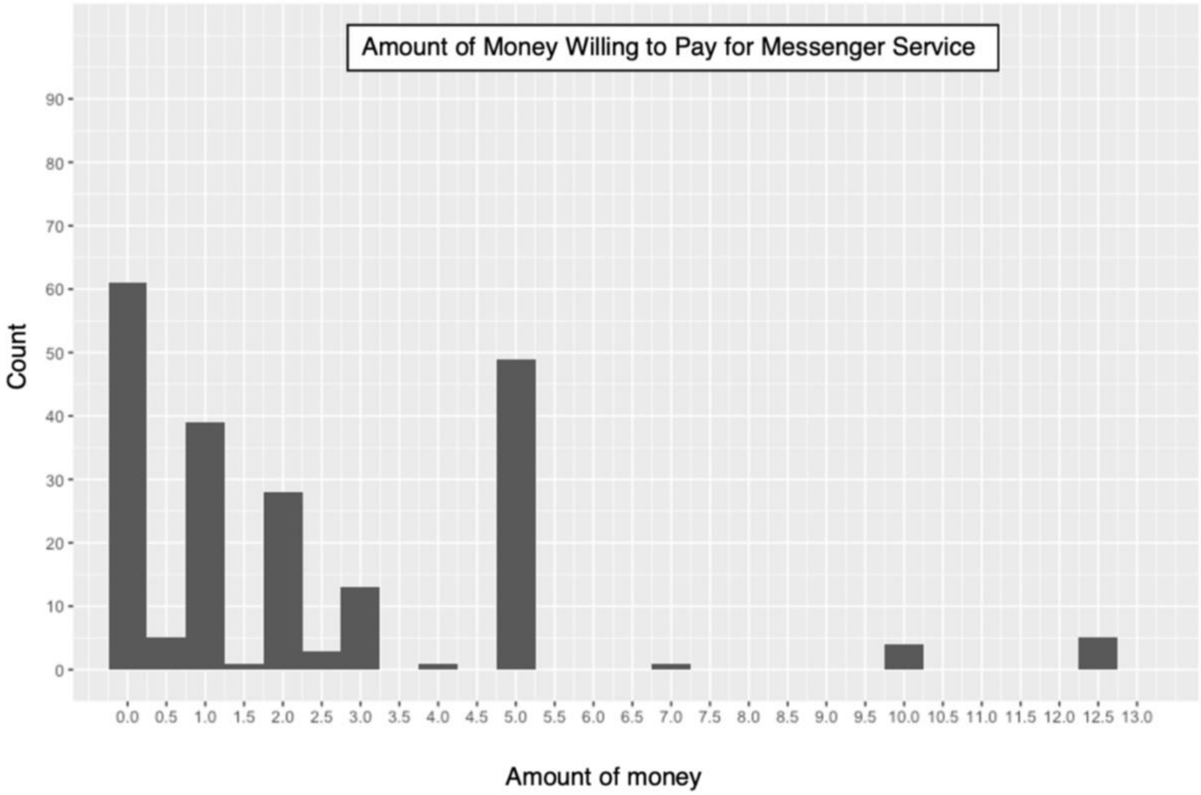

Supplementary Figure 3. Histogram of the variable on how much money participants would be willing to pay per month for a single messenger service; the x-axis shows the amount of money and the y-axis the number of participants indicating to be willing to pay the respective amount of money (after dealing with univariate outliers).

Supplementary Table 2.
Distribution of how much money participants would be willing to pay per month for a single messenger service (after dealing with univariate outliers)

€	0.00	0.30	0.50	1.00	1.50	2.00	2.50	2.99	3.00	4.00	5.00	7.00	10.00	12.50
count	61	1	4	39	1	28	3	1	12	1	49	1	4	5

4 Correlations between the Big Five and each Item on whether Participants were Willing to Pay for Social Media

4.1 Descriptive Statistics

Supplementary Table 3.
Descriptive statistics of each item on whether participants were willing to pay for social media in the total sample (*N*=210)

	Marketing	Data Protection	Prolongation of Online Time	Fake News and Radicalization
M (*SD*)	2.54 (1.22)	2.67 (1.29)	2.40 (1.25)	2.98 (1.41)

Note: Marketing: "[…] if thereby it is ensured that my data accrued there are not used for marketing purposes", Data Protection: "[…] if thereby it is ensured that my data accrued there are better protected", Prolongation of Online Time: "[…] if thereby it is ensured that the social media offers are designed in a way that does not aim to prolong the time users spend online", Fake News and Radicalization: "[…] if thereby it is ensured that the problem of fake news and radicalization is reduced". Friedman test statistics: $\chi_2(3)=58.93$, $p<.001$; significant differences were found between each pair of items according to Wilcoxon Signed-Rank tests (all *p*-values < .029).

4.2 Correlations

Supplementary Table 4.
Correlations between the Big Five and each item on whether participants were willing to pay for social media

	Marketing	Data Protection	Prolongation of Online Time	Fake News and Radicalization
Openness	r_s=0.13, p=.068	r_s=0.10, p=.136	r_s=0.14, p=.041	r_s=0.09, p =.184
Conscientiousness	r_s=-0.04, p=.591	r_s=-0.02, p=.775	r_s=-0.13, p=.066	r_s=-0.04, p=.588
Extraversion	r_s=0.02, p=.808	r_s=0.01, p=.915	r_s=-0.00, p=.961	r_s=-0.05, p=.508
Agreeableness	r_s=0.21, p=.002	r_s=0.11, p=.122	r_s=0.18, p=.009	r_s=0.07, p=.320
Neuroticism	r_s=-0.07, p=.344	r_s=-0.08, p=.241	r_s=-0.00, p=.965	r_s=0.02, p=.800

Note: Spearman correlations corrected for age are presented in this table. Marketing: "[…] if thereby it is ensured that my data accrued there are not used for marketing purposes", Data Protection: "[…] if thereby it is ensured that my data accrued there are better protected", Prolongation of Online Time: "[…] if thereby it is ensured that the social media offers are designed in a way that does not aim to prolong the time users spend online", Fake News and Radicalization: "[…] if thereby it is ensured that the problem of fake news and radicalization is reduced".

Supplementary Table 5.
Correlations between the Big Five and each item on whether participants were willing to pay for social media in males and females separately

	Males (n=117)				Females (n=91)			
	Marke-ting	Data Protec-tion	Prolonga-tion of Online Time	Fake News and Radica-lization	Marke-ting	Data Protec-tion	Prolonga-tion of Online Time	Fake News and Radica-lization
Openness	r_s=0.15, p=.106	r_s=0.12, p=.216	r_s=0.19, p=.044	r_s=0.08, p=.366	r_s=0.09, p=.374	r_s=0.10, p=.369	r_s=0.11, p=.324	r_s=0.09, p=.423
Conscientiousness	r_s=-0.01, p=.880	r_s=-0.00, p=.994	r_s=-0.14, p=.144	r_s=-0.08, p=.366	r_s=-0.04, p=.715	r_s=-0.03, p=.750	r_s=-0.11, p=.316	r_s=-0.01, p=.930
Extraversion	r_s=0.06, p=.528	r_s=0.06, p=.520	r_s=0.08, p=.370	r_s=-0.03, p=.777	r_s=-0.08, p=.439	r_s=0.08, p=.445	r_s=-0.14, p=.193	r_s=-0.13, p=.211
Agreeableness	r_s=0.19, p=.041	r_s=0.12, p=.198	r_s=0.26, p=.004	r_s=0.07, p=.435	r_s=0.22, p=.034	r_s=0.09, p=.422	r_s=0.11, p=.318	r_s=0.03, p=.749
Neuroticism	r_s=-0.11, p=.249	r_s=-0.07, p=.425	r_s=-0.02, p =.836	r_s=0.08, p=.374	r_s=-0.08, p=.476	r_s=-0.14, p=.193	r_s=-0.01, p=.902	r_s=-0.12, p=.272

Note: Spearman correlations corrected for age are presented in this table. Marketing: "[…] if thereby it is ensured that my data accrued there are not used for marketing purposes", Data Protection: "[…] if thereby it is ensured that my data accrued there are better protected", Prolongation of Online Time: "[…] if thereby it is ensured that the social media offers are designed in a way that does not aim to prolong the time users spend online", Fake News and Radicalization: "[…] if thereby it is ensured that the problem of fake news and radicalization is reduced". Two participants stated "third gender" as their gender identity and are not included in this table due to the low number of individuals in this group.
